# Hospital and Institutional News

**Published:** 1920-03-13

**Authors:** 


### HOSPITAL AND INSTITUTIONAL NEWS.

### THE R.A.M.C. MEMORIAL.

A satisfactory committee having been formed to organise a suitable war memorial to the R.A.M.C., a large meeting at which Ireland, Scotland, England and Wales were all represented was recently held, and the following decisions arrived at. (*a*) That a permanent memorial should be erected in London, with replicas in Edinburgh and Dublin, if funds were available. (*b*) That the names of officers, non-commissioned officers and men who had fallen in the war should be inscribed on the memorial. (*c*) That funds "ear-marked" for the benefit of families should be devoted to that purpose. (*d*) That it should be open to the public to subscribe such sums as they wish, and that these subscriptions should be sent to the Hon. Secretary, R.A.M.C. War Memorial Fund, Captain A. R. Wright, D.S.O., War Office, Cornwall House, Stamford Street, S.E. 1.

### RADIOLOGICAL RESEARCH.

In commemoration of the work and sacrifice of the late Dr. C. R. C. Lyster, whose death we had recently to deplore, the Senate of the University of London have instituted a Chair of Radiology at the Middlesex Hospital. This follows closely upon the appointment of a Professor of Physics at the hospital, and it is hoped that a combination of work by these two departments will produce unprecedented opportunities for developing new methods of diagnosis and treatment of disease. The establishment of the Chair is, however, but the mere beginning and, as with the expenses incurred in all complicated scientific investigation, adequate financial support is both essential and urgent. The Middlesex Hospital authorities are confident that invaluable research work can be done by their new units, and we cannot urge too strongly the essentially deserving nature of their appeal. Radiology and electrophysical medicine generally is in a stage from which the greatest human benefits may ultimately arise. Already teaching of the subject to the younger medical generation is better organised, and the whole movement is uncommonly well directed.

### ANOTHER MEMORIAL.

It is of interest also to note that it is proposed to found a Chair of Radiology as a memorial to Sir James Mackenzie Davidson, whose experimental work on *x*-rays application to practical medicine has been of such outstanding value. An appeal for funds is headed by Mr. Bonar Law and supported by leading men in the medical and scientific professions. Industry, too, which has in very many ways benefited from Sir James's work, joins in the suggestion. It is hoped, if sufficient funds be forthcoming, to found an institute for both teaching and research. Cheques should be made payable to the London Joint City and Midland Bank, marked "The Mackenzie Davidson Memorial Fund," and forwarded to Dr. Robert Knox, 38 Harley Street, W. 1.

### THE INFLUENZA OUTLOOK.

From recent official publications and from overseas news it may probably be concluded that influenza is undoubtedly epidemic in a few localised English and Welsh communities, and also that, though of distinctly lower virulence, the type is similar to that of 1918-19. This is in marked contrast to the situation in several American and European Continental cities, and therefore may we congratulate ourselves upon our good fortune. Nevertheless, without in any sense advocating panic measures, we would urge both the people and the press to take the warnings of the Ministry of Health with all seriousness. Authorities are not only anxious that the possibilities should be realised, but, more important, that organisation and preparation should be complete *before* the appearance of the disease in serious epidemic form. This calamity may, indeed, not occur; but forewarned should mean fore-armed and in their precautions the Ministry of Health deserve the full sympathy and co-operation of the country.

### A NEW JOURNAL.

We have to welcome the recent appearance of a first number of the *British Journal of Experimental Pathology*, and apart from the fact that this branch of science has, in the past, been relatively inadequately represented by a press of its own, there is considerable satisfaction to be derived from the news that the editors of the newcomer construe pathology in its broadest sense. Experimental work in bacteriology, physiology, and biochemistry are all to be dealt with, providing the particular investigation has a direct bearing upon our knowledge of disease. Every effort will apparently be made to keep in touch with and also to summarise valuable work of this nature which may be done overseas and, in that experimental pathology, when all these fields are included in the term, has now become one of the most prolific sections of medical research, the new journal should have before it, not only an eminently successful career, but should fill a gap in British scientific literature which has of late become progressively more obvious.

### THE ARBITRATOR'S AWARD.

The Ministry of Health announce the award fixed by the arbitrators, W. F. Gore Browne, K.C., Sir R. V. Vassar-Smith, and Dr. J. C. Cramp, on the question of practitioners' remuneration under the Insurance Acts, to be a capitation fee per insured person at the rate of 11s. per annum. In forming this conclusion due account has been taken of the agreement that the capitation fee is not to include any payment in respect of the supply of drugs and appliances, nor any payments to meet those special conditions of practice in rural and semi-rural areas which are covered by the payments to be made out of the Central Mileage Fund.

### A PLAN OF CAMPAIGN FOR THE THREE FUNDS.

On March 5, *The Hospital Saturday Fund Journal*, the enlargement of which we have already welcomed, suggests a plan of campaign to relieve the London hospitals of their debt. In an editorial thereon it is well said that to supersede the voluntary system by State hospitals would be a calamity the extent of which would not be realised "till too late." Many will never fully realise their value until they have disappeared, and then regrets will be idle. The writer, adopting a correspondent's suggestion, believes that the various appeals now published create confusion, and that often the public sympathy is so much distracted that gifts are not made at all. He urges a joint effort by the three great funds for the benefit of all the London hospitals, an effort which he suggests should take the form of at least a week's campaign to begin on a Sunday in the churches and to be pursued throughout the week in streets, factories, workshops, retail stores, and perhaps the tube railways. A weekday should be given to each of these, and the campaign should end with a final effort on Saturday in all places of amusement, playing-fields, and sports grounds. It is an intelligible suggestion, and a meeting of the three funds should be held to discuss it. Our only criticism concerns the proposal to pay all the proceeds into a central fund. Would it not be simpler to entrust the King's Fund with the proceeds, and that it should be directed to distribute them at once, apart from its own receipts, through a committee on which the two other funds should be represented?

### EMPLOYERS' CONTRIBUTIONS: AN INTERESTING EXTENSION OF PRINCIPLE.

We are glad to see that the organisation of Employers' Subscription Funds, of which the Leeds scheme, already published fully in The Hospital, is a model, is making progress generally, especially in the cotton trade, where profits have been immense and hospital subscriptions very little organised. A modest plan has been started at Ashton, where the members of the Ashton and District Cotton Employers' Association have recommended every member to contribute ¼d. per spindle, male equivalent, including looms, towards a special fund to pay the debts of the Ashton District Infirmary. This contribution will be in addition to an annual subscription at the rate of 5s. per thousand spindles. If our mathematics are not at fault, this means that the donation to the special fund will be at the rate of approximately £1 per thousand spindles. It is also an interesting extension of principle. At last employers have learnt that their subscription should not be a casual sum, but, in decency, should bear some relation to the number of men in their employment. This is the organisation of subscriptions. It is something new to apply the same principle to donations, to have them also on a reasonable plan. We applaud the development, and invite other centres to follow the example set at Ashton, which sets a precedent that can be quoted.

### THE JACKSON BABY: A DISGRACEFUL CASE.

The general protest which was raised a few years ago against the introduction of the words "no encumbrances" into advertisements of vacant posts under the Poor Law, is likely to be raised even more vigorously again. A child, now seventeen weeks old, was born to the porter and the portress of Bramley Union, Yorkshire. A few weeks since the Bramley Board of Guardians passed the following resolution: "That in the best interests of the institution and of the discipline, and as well for the future benefit of the child, it is desirable that Mr. and Mrs. Jackson should make preparation for placing out their child under proper care, to be maintained outside the institution, the same to take effect not later than March 1." Mr. Jackson resolutely refused to be deprived of his child, whereupon he and his wife received one month's notice. Pursuing the same policy, the guardians also dismissed Mr. and Mrs. Parker, two attendants, because they had a child of three living with them. The Yorkshire District Poor Law Workers' Union has taken up the question, which the president, Mr. E. Halford, of St. Luke's Hospital, Bradford, rightly describes as a national one. The Clerk to the Bramley Union is Mr. A. Gaunt, who is the guardians' instrument in this disgraceful action. We challenge him and his Board to provide any reasons for this attempt to deprive a married couple of their child, which the public conscience will not repudiate. At a time when Poor-law Guardians are endeavouring to justify their survival, and to claim that the Poor Law is humane and sympathetic, such a case as this gives these professions the lie. If people wish to realise the spirit which inspires public institutions, the sort of conscience which guides the managers of rate-supported institutions, the Jackson case is a witness to the truth. We invite Dr. Addison's attention to this matter, and the public should insist upon knowing what view is taken by the Ministry of Health of the action of the Bramley Guardians. Mr. Jackson is to be congratulated upon his refusal, and he will have the support of every decent person in defence of his elementary right to the company of his own child.

### THE LONDON AND COUNTIES MEDICAL PROTECTION SOCIETY.

The annual report and accounts of this medical protection society are issued, with commendable celerity, for the year ending December 31, 1919. On the financial side steady progress is shown; for the Society ended last year nearly two thousand pounds to the good over the previous year. We note, however, that on the ordinary income and expenditure account the Society just failed to make ends meet, and that although there was a satisfactory surplus on the insurance (indemnity) fund account, yet it was a good deal smaller than usual owing to claims against the fund of much more than ordinary magnitude. The only other noteworthy point is an item in the agenda for the annual meeting (on March 17) to "consider a proposal for the remuneration of directors attending the meetings of

Council." We have no information as to who constitute the "Directors" of the Society, nor as to what the proposed remuneration amounts to. If it is merely the payment of train fares and other out-of-pocket expenses—as is the custom with the Medical Defence Union, we believe—of country members of the Council, the suggestion will probably command asent; but if it means payment of all or some of the Council for their attendance at meetings, it constitutes a new principle of great importance, and one we should be very sorry in many ways to see adopted.

### HOSPITAL STRIKE AT JOHANNESBURG.

For some time past, writes our South African correspondent, there has been much unrest and trouble among the natives—over a hundred in number—employed at the Johannesburg General Hospital. They had pressed for increased wages and for reduced hours. The superintendent gave them shorter hours in January last, and the House Committee recommended an increase of 5s. per month to those natives getting less than £3 10s. per month. The Finance Committee did not agree to this recommendation, and the natives in consequence struck work on February 4. The assistance was called in of Colonel Pritchard, Director of Native Affairs, who addressed the native strikers and ultimately persuaded them to return to work.

### THE PRUDENTIAL ASSURANCE COMPANY.

The seventy-first annual report of the Prudential Assurance Company, recently issued, is a document which is worth the study of treasurers and members of finance committees of voluntary hospitals. Not because it deals in millions—nay, hundreds of millions—but because it illustrates that sound and sane and safe commercial policy which underlies the Prudential's vast and almost terrifying success. For instance, the directors have "written down" the book values of their investments this year by no less than two and a-half millions of pounds; mainly, one may suppose, it is the Russian, German, and Austrian securities to which this drastic process has been applied. The report shows continuous and gratifying expansion of all sections of the Company's business, and both policy-holders and shareholders better than ever entrenched behind the calamity-proof protection of the Prudential's unique financial resources and apparently unassailable and inexhaustible prosperity.

### A HOSPITAL PRESIDENT'S BEQUEST.

The late Mr. John Sykes, formerly President of Huddersfield Royal Infirmary, has made a munificent bequest to the town. By his will he has presented to the Town Council the Hospital for Tuberculosis Children at Bradley Gate, which has now been handed over completely furnished and ready for occupation. On behalf of the executors, Mr. William Ramsden explained to the Town Council, at its recent meeting, that Mr. Sykes, when President of the Royal Infirmary, had been impressed with the need for treatment of tuberculosis, particularly among children. He therefore purchased nearly fifteen acres of land at Bradley Wood, and gave his executors the means to fulfil his intention. Including the price of the land, the sum spent on the children's hospital was about £12,000. The Mayor of Huddersfield, Mr. Woolven, gratefully accepted the gift on behalf of the Corporation, and the Health Committee has gladly undertaken to fulfil the wishes of the testator. The gift is remarkable not only because it was made by a former President of a voluntary hospital, but also because his private enterprise has endowed the Corporation with a hospital, though it has often been argued the local authorities have long possessed sufficient legal powers to provide their districts with such hospitals as may be needed. The moral seems to be that private initiative is more generous and far-seeing than any public body—an old truism which it is the fashion nowadays to ignore.

### THE NORTHERN WOMEN'S HOSPITAL FOR PAYING PATIENTS.

The fact that there are only two large infirmaries in the Newcastle-on-Tyne and Sunderland areas to serve about three million people was referred to by Dr. Ranken Lyle, a practitioner in Newcastle, at the recent meeting of the Northern Women's Hospital, one of the latest hospitals of the district. The hospital was founded to provide accommodation for professional women and girls, the wives of clerks, etc., who are not eligible for admission to Newcastle Infirmary, but who cannot afford the expense of the existing nursing homes. Thus it serves patients suffering from women's complaints who dislike the publicity of a large hospital, whether they require surgical or medical treatment. During last year the hospital was constantly full. The cost for one year was reported by Miss Margery Taylor, the hon. treasurer, to have been £1,582, or £113 per bed. Receipts from patients for fees, drugs, dressings, and in donations, amounted to £662, or five-twelfths of the total costs. The annual subscription list has now reached £625 6s., which it is hoped to raise to £1,000. The matron, Miss Adcock, and the nursing staff were praised by Dr. Ranken Lyle, who remarked that he did not know a better conducted hospital. He hoped that the Committee would extend the institution. We should like further details of the administration, conditions of entry, and the fees charged.

### MR. J. KAY AND THE COTTON TRADE.

While the newspapers continue to bewail the "crisis" in voluntary hospital finance it is refreshing to turn to a hospital meeting where the opening speaker began by reminding his audience of the successful effort which has been made to relieve it at Cardiff, Nottingham, Bolton, Preston and Warrington. There is more hope in such reminders than in any multitude of wails. Of Leeds, Cardiff, Nottingham, we have already given some account; but we urge every hospital which is launching a new scheme successfully to send us a record of its proposals, so that, alone among the London newspapers, The Hospital may keep a firm front, and by a series of facts each week do something to check the wild assertions that are only being made as an excuse for destroying the voluntary system by the introduction of State control. Blackburn, which needs £100,000 to build a new wing at the Royal Infirmary, has some encouraging facts to set beside the others we have mentioned. Last year its income increased by £5,000; the scheme for weekly contributions has now been adopted by 400 mills and workshops; and the Mayor's committee hopes to announce shortly that every factory has joined this scheme. The prosperity of the Lancashire cotton trade owes a big debt to the hospitals which it can easily repay. Mr. J. Kay, we understand, is about to approach the trade and to ask it to combine in a plan which will raise £100,000, and so give to East Lancashire the hospital that it needs as the district's war memorial. The cotton trade should take the chief part in this, for the war has made it prosperous. Mr. Kay is a man of conviction, and we hope to hear from him again.

### A PLAN OF CAMPAIGN AT BOURNEMOUTH.

Under Mr. C. H. Cartwright, the Mayor of Bournemouth, the appeal for £20,000 on behalf of the Royal Victoria and West Hants Hospital is well on its way. As we write a good step towards half the sum has been already taken. Mr. Cartwright makes a special reference to the industrial classes of the town, and is appealing to employers to confer with their staffs so as to arrange for weekly collections. So far, so good. We hope, however, he will also start an employers' subscription fund, on the model of that successfully established at Leeds; and that he will remember that the principle of sectional subscriptions applies not only to capital and labour, but to every class. Now Bournemouth is rich in residents and in visitors. We suggest, therefore, that he should call a meeting of residents and appeal to their public spirit to organise a Bournemouth Residents' Fund, the members of which would agree to contribute annually. The class of annual subscribers does not cohere because it is not organised. It should be organised or have regular meetings at which qualified speakers, perhaps outsiders of distinction, should lecture on hospital life and work, and expound the doctrine of personal service which lies at the basis of the voluntary principle. This would weld the residents into a corporate body, with the hospital for their common centre. The visitors are another problem. But at each season during the year a hospital week should be devoted to them. Mr. Cartwright well knows the right way, and we are sure will realise the importance of a comprehensive plan of campaign.

### SOME INTERESTING OLD MEDICAL BOOKS.

At the sale of a portion of the Earl of Pembroke's library, which takes place on March 15, 16, and 17, at Messrs. Sotheby's rooms in New Bond Street, a good many ancient volumes of medical interest are included in the catalogue. Ulrich Hutten's black letter treatise (Lot 249) "of the wood called guaiacum that healeth" all manner of diseases, from stone to syphilis, is dated 1540. Lot 251 contains twelve pieces bound in one volume: one is "a corner stone laid towards the building of a new colledge, that is to say a new body of Physicians in London" (1675); and another—"I am for you all, Complexions castle" (1604)—perhaps emanates from some Elizabethan beauty specialist of a type that the "new colledge" later in the century was little likely to encourage. Lot 280 contains a 1676 treatise on Rules of Health; and Lot 449, dated 1613, tells how to cure the diseases produced by "the biting of mad dogges." Lot 451 is a "compendious or briefe examination of certayne ordinary complaints of divers of our countrymen in these our dayes," by William Stafford. "These our dayes" were the spacious times of Queen Elizabeth, for the volume was published in 1581. The bibliophile physician, if the breed is not extinct, should not miss this interesting sale.

### TYPES OF SOUTH AFRICAN PATIENTS.

The Administator of Natal (Mr. G. T. Plowman, C.M.G.) opened a new wing of Grey's Hospital, Pietermaritzburg, Natal, in December. The admissions to the hospital during the year ended September 30, 1919, numbered 2,331. Of these, 1,341 were Europeans; 234 Asiatics; and 756 natives. The hospital was established 65 years ago, and has increased until there are now 200 beds. There has been lately added a maternity block where midwifery training is pursued. The block consists of fifteen beds for Europeans and four for coloured people. The original hospital was named "Grey's Hospital" after Sir George Grey, then High Commissioner of South Africa and Governor of the Cape. It cost £2,000. The new wing has cost £18,000! The Administrator stated in the course of his address, that the annual Union expenditure on hospital maintenance has increased from £25,000 to £60,000. The demands for accommodation are increasing from day to day. These figures represent upkeep only. The Administrator closed up properly with an appeal for private endowments. In this connection a public subscription fund is being well supported in the City for a Children's Ward in memory of the late Dr. Buntine, who was drowned in the torpedoed "Galway Castle."

### THIS WEEK'S DRUG MARKET.

Much the same features characterise the drug market as have been prominent for the past few months; that is to say, the demand for export continues to be on a large scale, with the result that a severe tax is put on supplies and prices generally tend upwards. Very little ipecacuanha is to be found in London and high prices are asked for anything available. Ergot of rye is scarcer than ever and dearer. The price of glycerin has been again advanced. Cape aloes is in plentiful supply but curaçao is scarce and dearer. Other articles whose prices have advanced include citric acid, hexamine, thymol, coca butter, caffeine, emetin, eserin, aloin, apiol and atropine. Among those whose prices tend upwards are phenacetin, phenazone, formaldehyde and aspirin. Potassium bromide is coming in more freely from Germany and prices are depressed in consequence.

